# Association between food insecurity and mental health outcomes among a convenient sample of Lebanese pregnant women

**DOI:** 10.1371/journal.pone.0332581

**Published:** 2025-09-29

**Authors:** Rana Rizk, Maha Hoteit, Maroun Khattar, Yonna Sacre, Toni Sawma, Myriam El Khoury-Malhame

**Affiliations:** 1 Department of Nutrition and Food Science, School of Arts and Sciences, Lebanese American University, Byblos, Lebanon; 2 Institut National de Santé Publique, d’Épidémiologie Clinique et de Toxicologie-Liban (INSPECT-LB), Beirut, Lebanon; 3 PHENOL Research Group (Public Health Nutrition Program-Lebanon), Faculty of Public Health, Lebanese University, Beirut, Lebanon; 4 Department of Primary Care and Population Health, University of Nicosia Medical School, Nicosia, Cyprus; 5 Department of Nutrition and Food Sciences, Faculty of Arts and Sciences, Holy Spirit University of Kaslik (USEK), Jounieh, Lebanon; 6 Department of Psychology and Education, Psychology Program, School of Arts and Sciences, Lebanese American University, Byblos, Lebanon; American University of Beirut Medical Center, LEBANON

## Abstract

Food insecurity (FI) is a pressing public health challenge, suggested to be associated with psychological distress and detrimental effects, especially in vulnerable populations such as pregnant women. To date, little is known in Lebanese pregnant women on the association between FI and emotional and behavioral outcomes. Thus, this study aimed to explore the association between FI, and emotional (anxiety, depression, distress) and behavioral (disordered eating, sleep quality) outcomes in a convenient sample of adult Lebanese pregnant women. A cross sectional study involving 146 pregnant women was conducted between 20 January 2023 and 16 September 2024. An online questionnaire was used to collect sociodemographic, financial, and medical characteristics. FI was assessed using the Arabic validated version of the Household Food Insecurity Access Scale, anxiety and depression using the Arabic validated version of the Patient Health Questionnaire, distress using the Beirut Distress Scale, disordered eating using the Arabic validated version of the Disordered Eating Attitudes in Pregnancy Scale, and sleep quality using the culturally-adapted Arabic version of the Pittsburgh Sleep Quality Index. Enter logistic regression models assessed the determinants of the dependent variables: anxiety, depression, distress, sleep quality, disordered eating. Findings showed that 66.4% of participants had FI, 50.7% had anxiety, 45% had depression, 83.6% reported high distress levels, 9.6% had disordered eating, and 57.5% had poor sleep quality. FI was associated with higher distress level, disordered eating, and poor sleep quality. No associations between FI and anxiety and depression were found. Given the high levels of FI and psychological distress in our sample, we emphasize the need for a comprehensive approach to support the physical and psychological health of pregnant women in Lebanon, with a focus on addressing underlying factors such as FI. Antenatal care must prioritize assessing food security and screening for and treating associated mental and behavioral health problems.

## Introduction

Pregnancy is a critical stage during which the mother’s nutrition and mental health affect the offspring’s short-term development and long-term health and wellness [1,2]. This period is marked by physical, psychological, and hormonal changes. These changes are part of the physiological adaptation to pregnancy to support the mother’s needs and to respond to the development of the fetus [3]. These changes may increase pregnant women’s risk of developing mental health issues [4] including anxiety, depression, sleep disturbances, and disordered eating [5–8]. Maternal exposure to psychological distress during pregnancy is associated with impaired brain development and long-term neurobehavioral disorders in the offspring [9], as well as adverse outcomes for the pregnant women, including pre-eclampsia, pre-term delivery and spontaneous abortion [9].

Psychological distress, which represents a catch-all term for a variety of prevalent psychological conditions, ranging from subclinical symptoms to clinical diagnoses of anxiety, depression, stress, or posttraumatic stress disorder (PTSD), may be a sign of common mental illnesses or of poor mental health [10]. Nowadays, anxiety, depression, and stress are the leading mental health problems that cause disability globally [11]. Anxiety is defined as the body’s response to a perceived threat. It is often triggered by the feelings, thoughts, and beliefs of individuals. Depression is a condition characterized by a loss of pleasure or interest, guilty feelings, sadness, low self-worth, suppressed sleep quality or appetite, and being extremely tired. It impacts job’s performance, sleep quality, productivity, and interferes with daily activities. As for stress, it manifests as feeling that one’s demands exceed their capabilities [11]. In short, psychological distress interferes with the affected individuals’ feelings, cognition, and social abilities, and can lead to reduced productivity and unemployment.

Food insecurity (FI), or the lack of access to enough nutritious and safe food to support normal and healthy life [12,13], is one the most challenging global public health problems, affecting in 2023 around 2.33 billion people, with a steady prevalence since 2020 [14]. Specifically, in the Arab region, around 53.9 million people suffered from severe FI in 2021, reflecting an increase by 55% since 2010 [15]. The case is similar in Lebanon, an Arab country in which 47.3% of households experienced FI in 2022 [16], due to accumulating protracted adversities in the country since 2019 including: the economic crisis leading to the devaluation of the local currency, the increased prices of food items especially animal-based products, the COVID-19 pandemic, the Beirut port explosion which disrupted Lebanon’s ability to import food typically accounting for 80% of the nation’s food supply and the Syrian refugees crises [17].

The aforementioned adversities simultaneously unsettled the Lebanese population’s food security and mental health; these effects are potentially more pronounced in vulnerable populations, particularly pregnant women. For instance, FI goes beyond quantity and quality of food and is suggested to disrupt people’s mental health due to its direct relation with social, psychological, cultural, and physical dimensions [18]. In general, people with FI are more likely to have symptoms of stress, anxiety, and depression compared with food secure people [19]. Specifically, among pregnant women, the available scarce evidence indicates that pregnant women with FI who do not get enough or good sleep may be more likely to experience negative health effects [20]. Furthermore, FI may be associated with a release of stress hormones, which have been linked to pre-term birth as well as unhealthy changes in a mother’s pregnancy weight, disordered eating during pregnancy, as well as post-partum depression [21]. Nevertheless, to date the topic of FI and mental health outcomes in pregnant women remains underexplored and is of potential cross-cultural difference. For instance, in Ethiopia, pregnant women with FI showed significantly higher depressive symptoms and mental distress compared with their counterparts [22,23]. In North America, a systematic review and meta-analysis revealed that FI is associated with many maternal mental health outcomes, particularly depression and depressive symptoms, and anxiety-related outcomes [24]. In spite of the consistent association between FI and psychological discomfort in literature, the degree of this association varies greatly according to contextual and geographical factors [25]. This suggests that more studies on this topic are needed to cover the different geographic locations and cultures to come up with policies and interventions tailored to the specific populations and regions of interest.

In Lebanon, studies assessing the association between FI and mental health outcomes reported that FI is associated with poor mental health outcomes among university students [26], and with increased risk of depression among mothers [27], highlighting the importance of assessing FI in vulnerable populations. However, to date little is known in Lebanese pregnant women on the association between FI and mental health outcomes, including anxiety, depression, and distress, as well as behavioral outcomes, including sleep and disordered eating, especially between the years 2019 and 2024. For instance, the ongoing crisis has put the health of Lebanese pregnant women under threat, as it led to increased reliance on the cheapest alternatives, or even skipping meals [28], heightening Lebanese pregnant women’s risk of psychological distress and disordered eating [29]. Recently, 87.8% of Lebanese pregnant women were found to experience mild to severe depression and 70.3% had significant anxiety levels [30], highlighting the importance of targeting mental health during pregnancy and identifying its predictors. Moreover, FI is suggested to be associated with poor sleep quality [31], and this has detrimental effect on the mother and the fetus. For instance, poor sleep quality is linked to several pregnancy complications, such as extended labor, pre-eclampsia, and gestational diabetes. Additionally, poor sleep quality might lead to pre-term delivery, low birth weight, and intrauterine growth retardation [32]. Understanding these intricate connections could better inform targeted policies and interventions to enhance and prioritize food security profile among Lebanese pregnant women, and subsequently decrease their psychological distress levels, and eventually improve overall pregnancy and offspring outcomes. Moreover, understanding FI among pregnant women and families with young children would be crucial in meeting the 2030 Sustainable Development Goals with a notable focus on enhancing maternal and child nutrition. As such, our study intended to explore the association between FI and general emotional states, anxiety, depression, and distress, as well as key behavioral outcomes, sleep quality, and disordered eating in a convenient sample of pregnant women in Lebanon.

## Materials and methods

### Study design

A cross sectional study which involved a convenient sample of Lebanese pregnant women (N = 146) was conducted between 20 January 2023 and 16 September 2024. Eight participants did not consent and exited the survey (94.8% response rate).

### Sample size calculation and sampling technique

The sample size calculation was conducted using the “G*Power, version 3.1”, based on similar existing literature [33] reporting an adjusted odds ratio of 1.8 for the association between perceived stress and food insecurity status among pregnant, and a 5%-type I error, the sample size needed is 150 participants.

### Ethical considerations

The study protocol followed the guidelines laid by the Declaration of Helsinki and was approved by the Institutional Review Board (IRB) at the Lebanese American University (LAU) (LAU.SAS.RR2.20/Jan/2023).

### Study population

Pregnant women who met the following eligibility criteria were included in the study: Lebanese, adult, residing in Lebanon, able to read English or Arabic, and able to use Google Forms to complete the survey. Women excluded from the study were those with medical problems and/or severe gestational complications such as placental abruption, pre-eclampsia (i.e., hypertension with proteinuria) and eclampsia, and maternal thrombosis.

### Data collection

Data were collected using an online questionnaire (Google Form), available in both English and Arabic (S1 Supplementary Material). The participants were asked to choose their preferred language to complete the questionnaire. Informed consent was obtained from participants via an approval button on the first page of the Google Form. When available, validated tools in Arabic language were used while non-validated tools were translated to Arabic using simple Arabic language to accommodate varying education levels. The study tool was pilot tested among 5 pregnant women.

### Sociodemographic, financial and pregnancy characteristics

The online survey also included questions regarding sociodemographic characteristics (age, residency, setting, education level, crowding index, smoking, employment status, and financial independency) of participants. In addition, pregnancy information were collected and included pregnancy trimester, parity, the use of supplements, pregnancy complications (anemia, hypertension without proteinuria, gestational diabetes, constipation), sex of the fetus, number of fetuses, primary caregiver, and pre-pregnancy weight and height.

### Food security status

Food security status was assessed using the Arabic validated version of the Household Food Insecurity Access Scale (HFIAS) [34]. This tool is used in many countries and comprises nine questions asked with a recall period of four weeks which allow to differentiate food secure from food insecure households across distinct cultural backgrounds. The score ranges from 0 to 27 and a higher score indicates that a household is experiencing higher FI [35]. Besides calculating the score, participants were also classified into four categories of FI (with food security, with mild FI, with moderate FI, or with severe FI) according to Coates et al. [35].

### Anxiety and depression

Anxiety and depression were assessed using the validated Arabic version of the Patient Health Questionnaire (PHQ-4) [36]. PHQ-4 is a tool that assesses simple severity of anxiety and depression rather than being a clinical diagnostic tool, with scores above six indicating high likelihood of clinical diagnosis. This tool is composed of four questions assessing the frequency of experiencing symptoms of depression and anxiety in the past 4 weeks. The first two related to anxiety and the last two to depression. Sub-scales of anxiety and depression are calculated, whereby a score of 3 or greater on each subscale is considered positive for screening purposes [36].

### Distress

Distress was assessed using the Beirut Distress Scale (BDS-10), which is validated to be used in the Lebanese population and includes 10 items exclusively related to stress, and measures stress level over the past week. A higher score reflects a higher distress level [37].

### Disordered eating

Disordered eating was assessed using the Disordered Eating Attitudes in Pregnancy Scale’s (A-DEAPS) validated Arabic version in Lebanon. This is a 10-item tool used to assess disordered eating attitudes among pregnant women, such as worries about weight gain, calorie restriction and food anxiety. A-DEAPS is shown to be reliable to be used in the Lebanese population [38]. Score ranges from 0 to 10 and a higher score reflects more disordered eating during pregnancy [38]. In our study, we used the 90^th^ percentile as the cutoff for being at risk of disordered eating, based on previous literature [39].

### Sleep quality

Sleep quality was assessed using the Pittsburgh Sleep Quality Index (PSQi). This tool is designed to assess the overall sleep quality in different clinical populations and is composed of 9 self-reported items addressing sleep latency, subjective sleep quality, sleep efficiency, sleep duration, sleep disturbances, use of sleeping pills, and daytime dysfunction in the past month. A score of 5 or more denotes an overall poor sleep quality. PSQi has a diagnostic sensitivity of 89.6% and specificity of 86.5% (p-value<0.001) [40]. In our study, we used the culturally-adapted Arabic version of the PSQi [41].

### Adherence to the mediterranean diet (MD)

Adherence to MD was assessed using the Mediterranean Diet assessed using the Mediterranean Diet Adherence Screener (14-MEDAS) [42]. This tool is among the widely used screeners in this regard, globally and in Lebanon, and shows good internal consistency [43–48]. Possible score ranges from 0 to 14 whereby a score of ≤5 indicates weak adherence, 6–9 indicates moderate to fair adherence, and a score ≥10 indicates good or very good adherence.

### Data management and statistical analysis

Data were managed on Microsoft Excel then transferred to the “Statistical Package for the Social Sciences, the IBM SPSS Statistics 26” and analyzed at a 95% confidence interval. Descriptive statistics summarized quantitative variables using means and standard deviations as well as frequencies and percentage. The sample size was relatively high (n > 100); hence the normality of the data was not explored. Dependent variables (disordered eating, anxiety, depression, sleep quality, and distress) were dichotomized; HFIAS score was used as a continuous variable but reported as both continuous and categorical. Association between the dependent variables and HFIAS score was assessed using Pearson correlation test. Association between the dependent variables and categorical variables (age, residency, setting, education level, etc.) was assessed using Chi-square tests. Enter logistic regression models were performed to assess the determinants of the dependent variables at a 95% confidence level. Independent variables entered in the model were the ones informed by the literature and having a p-value<0.2 in the bivariate analysis. To avoid overfitting, the number of allowed confounders per model was 10% of the smallest group (S1 Table). An omnibus test was used to check the overall significance of the models. The results of the logistic regressions were reported as adjusted Odds Ratios (aOR) and their respective 95% confidence interval (95%CI).

## Results

### Characteristics of the study participants

The sociodemographic and financial characteristics of study participants are shown in Table 1. More than half of the participants were aged 30 years or less (54.5%); participants were almost equally distributed between rural (49.3%) and urban (50.7%) areas. The majority had a university degree or equivalent (62.3%); however, more than half were unemployed (58.2%), and 43.8% reported being financially dependent. Data were collected amid a financial and economic crisis in Lebanon; this could have partly led to these high unemployment rates. The majority were living in a non-crowded household (67.8%), potentially reflecting a higher socioeconomic for these participants compared with those living in a crowded one.

**Table 1:** Sociodemographic and financial characteristics of study participants.

Sociodemographic & Financial Characteristics		N	%
Age Category	<= 30 years	79	54.5%
	> 30 years	66	45.5%
Residency	Beirut/Mount Lebanon	68	46.6%
	Others	78	53.4%
Setting	Rural	72	49.3%
	Urban	74	50.7%
Education Level	University/Equivalent	91	62.3%
	Others	55	37.7%
Employment Status	Unemployed	85	58.2%
	Employed	61	41.8%
Crowding Index	Not Crowded (less than 1 person per room)	99	67.8%
	Crowded (1 or more person per room)	47	32.2%
Financially Independent	No	64	43.8%
	Yes	48	32.9%
	Prefer Not to Say	34	23.3%

Table 2 shows the pregnancy characteristics of the study participants. Most of the participants were in their second (38.6%) and third trimester (39.3%) of pregnancy. Almost half (49.3%) had a normal pre-pregnancy body mass index (BMI) while 45% had either overweight or obesity. About one third reported having anemia (32.2%) and around 45% reported having constipation. The majority reported taking iron (68.5%) and calcium (56.2%) supplements and less than one third reported taking folic acid (29.5%) and iodine (16.4%) supplements.

**Table 2:** Pregnancy characteristics of the study participants.

Pregnancy Characteristics		N	%
***Pregnancy Information***			
Pregnancy Trimester	First Trimester	32	22.10%
	Second Trimester	56	38.60%
	Third Trimester	57	39.30%
Parity	No	84	57.50%
	Yes	62	42.50%
Sex of the Fetus	Male	45	30.80%
	Female	54	37.00%
	Don’t Know	47	32.20%
Number of Fetuses	One	138	94.5%
	More than one	8	5.5%
Pre-pregnancy BMI	Normal	71	49.30%
	Underweight	7	4.90%
	Overweight	37	25.70%
	Obesity	29	20.10%
Caregiver	Private Physician	104	71.20%
	Other	42	28.80%
Breastfeeding	No	6	4.10%
	Yes, Mixed	35	24.00%
	Yes, Exclusive	94	64.40%
	Did not decide yet	11	7.50%
***Pregnancy Complications***			
Presence of constipation	No	79	54.10%
	Yes	67	45.90%
Presence of anemia	No	99	67.80%
	Yes	47	32.20%
Presence of hypertension	No	138	94.50%
	Yes	8	5.50%
Presence of gestational diabetes	No	63	43.20%
	Yes	8	5.50%
	Not Tested	75	51.40%
***Supplementation***			
Iron	No	46	31.50%
	Yes	100	68.50%
Calcium	No	64	43.80%
	Yes	82	56.20%
Vitamin B9	No	103	70.50%
	Yes	43	29.50%
Iodine	No	122	83.60%
	Yes	24	16.40%
Multivitamin	No	45	30.80%
	Yes	101	69.20%

### Food security status and mental health outcomes among study participants

The mean HFIAS score in our sample was 6.09 ± 7.01 and around two-thirds of participants (66.4%) had FI. Scores in our sample ranged from 0 to 27, and participants were distributed as follows: 33.6% with food security; 48.6% with mild FI; 8.9% with moderate FI; and 8.9% with severe FI. Mean and standard deviation (Mean±SD) for anxiety, depression, distress, sleep quality, and disordered eating were 3.02 ± 1.79, 2.69 ± 1.8, 15.76 ± 6.88, 6.75 ± 3.98, and 4.93 ± 2.98, respectively. Almost half of the sample had anxiety (50.7%) and depression (45%), with the majority reporting elevated distress levels (83.6%). More than half had poor sleep quality (57.5%), and few participants (9.6%) had a risk of disordered eating. Based on HFIAS, women with depression (***p-value**** = 0.014*), high distress (***p-value**** = 0.038*), poor sleep quality (***p-value**** = 0.006*), and at risk of disordered eating (***p-value**** = 0.014*) had a significantly higher FI score compared with their counterparts (**Table 3**).

**Table 3:** Mental health outcomes based on food security status among the study participants.

		N	%	HFIAS	*P-value*
				Mean±SD	
**Anxiety**	No anxiety	72	49.3%	4.99 ± 6.73	*0.061*
	Anxiety	74	50.7%	7.16 ± 7.16	
**Depression**	No depression	80	54.8%	4.80 ± 6.67	***0.014***
	Depression	66	45.2%	7.65 ± 7.15	
**Distress**	Moderate distress	24	16.4%	3.38 ± 5.03	***0.038***
	High distress	122	83.6%	6.62 ± 7.24	
**Sleep Quality**	Poor sleep quality	84	57.5%	7.45 ± 7.15	***0.006***
	Good sleep quality	62	42.5%	4.24 ± 7.43	
**Disordered Eating**	No disordered eating	132	90.4%	5.63 ± 6.56	***0.014***
	At risk of disordered Eating	14	9.6%	10.43 ± 9.60	

Values in **bold** indicate significant results.

### Association between food security status and mental health outcomes among pregnant women

As per **Table 4**, no associations between HFIAS and anxiety and depression were found. A higher HFIAS score was associated with higher odds of high distress level (**aOR:** 1.1, ***p-value**** = 0.033*), higher odds of being at risk of disordered eating (**aOR:** 1.11, ***p-value**** = 0.007*), and with lower odds of adequate sleep quality by 5.9% (**aOR:** 0.941, ***p-value**** = 0.045*).

**Table 4:** Regression models to assess association between food security status and mental health outcomes.

Model 1	Anxiety			
	aOR	95% CI		*p-value*
		Lower	Upper	
**HFIAS Score**	1.007	0.951	1.067	*0.806*
**Crowding Index**				
Not crowded (Reference)	–	–	–	*–*
Crowded	1.38	0.59	3.22	*0.454*
**Employment Status**				
Unemployed (Reference)	–	–	–	*–*
Employed	0.851	0.33	2.17	*0.735*
**Education Level**				
Intermediate/Secondary (Reference)	–	–	–	*–*
University	0.66	0.26	1.67	*0.386*
**Parity**				
No (Reference)	–	–	–	*–*
Yes	0.53	0.25	1.12	*0.099*
**Financial Independency**				
No (Reference)	–	–	–	*–*
Yes	0.44	0.15	1.27	*0.129*
Prefer not to say	1.04	0.42	2.62	*0.926*
**Presence of Anemia**				
No (Reference)	–	–	–	*–*
Yes	1.68	0.77	3.66	*0.193*
**Presence of Hypertension**				
No (Reference)	–	–	–	*–*
Yes	3.2	0.35	29.12	*0.303*
**Model 2**	**Depression**			
	**aOR**	**95% CI**		***p-value***
		**Lower**	**Upper**	
**HFIAS Score**	1.027	0.97	1.087	*0.35*
**Crowding Index**				
Not crowded (Reference)	–	–	–	*–*
Crowded	1.28	0.55	2.98	*0.565*
**Employment Status**				
Unemployed (Reference)	–	–	–	*–*
Employed	1.23	0.48	3.11	*0.666*
**Setting**				
Rural (Reference)	–	–	–	*–*
Urban	**0.44**	**0.21**	**0.92**	***0.03***
**Parity**				
No (Reference)	–	–	–	*–*
Yes	0.75	0.35	1.64	*0.477*
**Financial Independency**				
No (Reference)	–	–	–	*–*
Yes	**0.27**	**0.092**	**0.825**	***0.021***
Prefer not to say	0.63	0.25	1.59	*0.33*
**Presence of Anemia**				
No (Reference)	–	–	–	*–*
Yes	**2.47**	**1.1**	**5.52**	***0.028***
**Presence of Hypertension**				
No (Reference)	–	–	–	*–*
Yes	4.75	0.49	46.17	*0.179*
**Model 3**	**High Distress**			
	**aOR**	**95% CI**		***p-value***
		**Lower**	**Upper**	
**HFIAS**	**1.1**	**1.008**	**1.2**	***0.033***
**Setting**				
Rural (Reference)	–	–	–	*–*
Urban	**0.17**	**0.055**	**0.54**	***0.003***
**Presence of Constipation**				
No (Reference)	–	–	–	*–*
Yes	**5.04**	**1.6**	**15.92**	***0.006***
**Presence of Anemia**				
No (Reference)	–	–	–	*–*
Yes	**7.12**	**1.45**	**35**	***0.016***
**Model 4**	**Risk of Disordered Eating**			
	**aOR**	**95% CI**		***p-value***
		**Lower**	**Upper**	
**HFIAS**	**1.11**	**1.03**	**1.21**	***0.007***
**Crowding Index**				
Not crowded (Reference)	–	–	–	*–*
Crowded	**0.082**	**0.009**	**0.737**	***0.026***
**Pregnancy Trimester**				
First (Reference)	–	–	–	*–*
Second	1.16	0.29	4.56	*0.831*
Third	0.13	0.013	1.33	*0.185*
**Model 5**	**Determinants of Good Sleep**			
	**aOR**	**95% CI**		***p-value***
		**Lower**	**Upper**	
**HFIAS**	**0.941**	**0.88**	**0.99**	***0.045***
**Education Level**				
Intermediate/Secondary (Reference)	–	–	–	*–*
University/Equivalent	2.08	0.86	5.06	*0.106*
**Financial Independency**				
No (Reference)	–	–	–	*–*
Yes	1.009	0.39	2.61	*0.985*
Prefer not to say	**2.81**	**1.1**	**7.16**	***0.031***
**Presence of Anemia**				
No (Reference)	–	–	–	*–*
Yes	**0.4**	**0.17**	**0.92**	***0.031***
**Presence of Constipation**				
No (Reference)	–	–	–	*–*
Yes	**0.45**	**0.22**	**0.94**	***0.034***

Values in **Bold** indicate significant results. **Abbreviations: *aOR* A**djusted odds ratio, ***CI*** Confidence interval, ***HFIAS*** Household food insecurity access scale. **Variables entered in model 1:** HFIAS score, crowding index, employment status, education level, parity, financial independency, presence of anemia, presence of hypertension. **Variables entered in model 2:** HFIAS score, crowding index, employment status, setting, parity, financial independency, presence of anemia, presence of hypertension. **Variables entered in model 3:** Setting, HFIAS score, presence of constipation, presence of anemia. **Variables entered in model 4:** HFIAS score, crowding index, pregnancy trimester. **Variables entered in model 5:** HFIAS score, presence of anemia, presence of constipation, financial independency, education level.

## Discussion

To the best of our knowledge, this study is the first to assess the association between FI and emotional as well as behavioral outcomes in a convenient sample of pregnant women in Lebanon. The study thus adds to the body of literature on FI and psychological distress and lifestyle outcomes in times of unprecedented, protracted crises.

First, 66.4% of pregnant women in our sample had FI. Although estimates in low- and middle-income countries (LMICs) are quite variable, the overall levels remain nonetheless alarming. For instance, FI estimates range between 43.9% in Iran in 2017 [49], 67.4% in Ethiopia in 2021 [50], and reaching 75% in Nigeria in 2021 [51]. Lower prevalence of FI among pregnant women is systematically reported in high-income countries with 6.7% in the United States in 2020 [52], 12.8% in Canada [53], and 14.3% in Australia [54]. Our findings highlight the major worrisome discrepancy between low-middle-income countries and high-income countries, Importantly, our results suggest that FI may be more pronounced in Lebanese pregnant women compared with the general population’s FI estimates (47.3%) [16] thereby advocating the pressing need for immediate action to improve food security in this vulnerable population. The increased FI rates in Lebanon compared to other countires is due to the adversities faced by the country since 2019 (the economic crisis, the increased prices of food items, the COVID-19 pandemic, and the Beirut port explosion) [17].

In our sample, 50.7% of pregnant women were found to have elevated anxiety symptoms, 45% elevated symptomatology of depression and 84% of women reported being high distressed. Albeit not surprising given the overall unstable context in the country, these rates are alarming as mental disorders are known to have several negative outcomes in pregnancy for both mothers and babies. For instance, newborn biological indicators including height, weight, and head size are significantly impacted by extreme anxiety [55]. Prolonged or severe maternal anxiety may also alter the baby’s blood flow, making it more difficult for the developing organs to receive oxygen and other vital nutrients [55]. Additionally, pregnant women who suffer from chronic or severe anxiety may feel overburdened and exhausted, which may affect their sleep patterns, food choices, and regularity of prenatal care [55]. Regarding depression, high levels during pregnancy predict more serious complications such as post-partum depression, severely threatening mother and offspring’s health outcomes. For instance, small for gestational age, stillbirth, low birth weight, pre-term delivery, maternal morbidity such as perinatal problems, and increased surgical delivery are all positively correlated with maternal depression throughout pregnancy [56]. Additionally, stress leads to elevated cortisol levels and decreased food intake in mothers [57]. It is noteworthy that rates of depression and distress are significantly higher than those reported in other countries with global estimates with ranging from 9.5% to 27.85% [58–60] and 6 to 16.7% [57] respectively. These levels are nonetheless comparable with overall rates of stress in the country [61]. Our findings also showed that most pregnant women struggle with adequate sleep quality, and the odds of good quality were lower in participants with higher HFIAS score.

In our study, FI emerges as a chronic toxic stressor but not as a predictor of clinical mental illness. Although in our sample women with FI have higher levels of anxiety and depression, we found that FI does not directly associate with either anxiety or depression, while it acts as a determinant of overall distress and sleep alterations. FI is generally associated with negative mental health outcomes, including increased anxiety and depression [33,62–64]. The uncertainty and stress of not having reliable access to nutritious food exacerbates psychological distress [65]. However, some individuals may develop coping mechanisms or psychological adaptations to chronic FI, potentially leading to a blunted emotional response over time. This adaptation does not equate to a positive impact but rather reflects a complex interplay between chronic stress and mental health. It could be that in our sample high baseline anxiety and depression dilute the effect of FI due to ceiling effect. It could also be linked to psychometric properties of the scales as the PHQ4 scale is an ultra-brief and focused scale that might underestimates food-related triggers superseded by other predominant aspects in the Lebanese complex context (trauma, economic strain…), whereas the BDS and PSQi are more exhaustive and perhaps more culturally acceptable with easier to identify symptoms. It could lastly be that FI creates daily anticipation and leads to psychosocial distress (fear, shame, worry about feeding the children) and physiological hyperarousal (altered sleep) that may precede mental disorder states. The internalization of food-related worries in the form of clinical anxiety and depression diagnoses would be in turn buffered by factors such as the high education level of women in this sample and other strong family/social networks and religious coping in the country in general and in rural areas included in this study in particular. Education and social support are known protective factors againt mental distress even in dire situations [66]. Lastly, around 45% of women in this sample had pre-existing overweight or obesity. Their previous chronic exposure to weight stigma, FI, or body-related concerns may lead to psychological adaptation, numbing, or a form of coping desensitization, reducing the emotional impact of FI on mental distress while keeping it obvious for distress and behavioral aspects (sleep). It could conversely be that larger body size are culturally less stigmatized during pregnancy as pregnancy shifts the focus from body-weight to fetal health and obese women are transiently perceived as protective or nurturing [67]. FI might therefore be processed as less threatening to one’s health identity or maternal image among women with pre-pregnancy obesity, muting the psychological anxiety and depression usually associated with it.

In our study, the majority of pregnant women had no disordered eating; however, a higher FI score was associated with an increased likelihood of having disordered eating. FI was strongly associated with disordered eating in the United States [65], consistent with our findings. This is because the inability to get food may set off a chain of stressful events in the family setting, causing the mental health of the mother to deteriorate [68]. In addition, anxiety about accessing food might lead to skipping meals, and sometimes not eating the whole day [29]. Moreover, exposure to FI, even in environments with enough calories, has been proposed as a threat to one’s survival and well-being, increasing their stress levels and stress-induced eating [65]. Stress, for instance, when interpreted as a threat, triggers the hypothalamus-pituitary-adrenal axis, which in turn triggers a series of hormones, including leptin, insulin, cortisol, and neuropeptide Y, that have a direct impact on central fat storage, leading to increased weight and visceral fat accumulation during pregnancy [65].

Taken together, the current findings reveal a high prevalence of FI and psychological distress among Lebanese pregnant women. We further evidence significant associations between FI, psychological distress, and sleep alterations. This interconnected loop highlights the need for immediate sustainable strategies to reduce FI in Lebanon, especially among vulnerable populations like pregnant women. Pivotal steps must be implemented to improve resilience against crises, such as strengthening social safety nets, as a way of supporting the most vulnerable. In addition, investing in local agriculture can be used as an approach to decrease the FI burden and help populations avoid documented negative coping strategies [69], such as skipping meals or decreasing portion sizes, which have detrimental effect on maternal and fetal health [70,71]. When it comes to supporting pregnant women’s mental health, international and local non-governmental organizations as well as medical associations play a critical role, especially in times of crises. National mental health awareness campaigns, promoting mental health at workplace, providing high-quality mental health preventive and curative services, and community-based activities in healthcare centers targeting pregnant women, are all effective at reducing the burden of psychological distress among pregnant women, and improving their overall well-being and eventually pregnancy outcomes. In sum, pregnant women are advised to engage in regular physical activity, prioritize a healthy diet, and actively seek social support, such as attending antenatal classes, and openly communicate and share their feelings with their healthcare providers and family.

### Strengths and limitations

The present study has the strength of being the first-of-its-kind in Lebanon and the region to assess the association between FI and psychological distress among pregnant women. As such, this study adds to the literature by addressing a state of FI and mental health that is still under-explored. The findings can be used by policy makers as well as health workers to adopt targeted interventions that help improve the overall health and well-being of pregnant women in Lebanon. However, this study has some limitations. Being a cross-sectional study, it limits the ability to establish causality between FI and mental health outcomes, which means the study cannot determine whether FI leads to mental health issues or if mental health problems contribute to food insecurity. Plus, The use of a convenience sampling method may introduce selection bias, as the sample may not be representative of the broader population of pregnant women in Lebanon. This could affect the external validity of the findings. In addition, the data is self-reported and could be subject to response bias which can lead to inaccurate reporting of food insecurity and mental health symptoms, as well as social desirability bias, all of which can skew the results. Additionally, the study did not account for all potential confounding factors which may influence the observed associations. Moreover, the 14-MEDAS, used to assess MD adherence, is not validated in Lebanon, which might affect our results.

## Conclusions

Lebanese pregnant women exhibit high levels of FI and poor mental and behavioral outcomes; all of which have a heightened risk of negative pregnancy outcomes. FI was associated with an increased burden of disordered eating, increased likelihood of high distress, and decreased likelihood of good sleep in our sample. This study calls for a comprehensive approach to bolster altogether physical and psychological health of pregnant women in Lebanon by targeting its underlying components, particularly FI. Due to its known deleterious effects on maternal health and pregnancy outcomes, antenatal care in Lebanon must prioritize assessing food security status and screening for and treating associated mental and behavioral health problems. Monitoring food security regularly is vital for directing food and financial aid, supporting early famine warning systems, evaluating development, health, and nutrition activities, and driving government policies across different sectors [72]. In this context, and as evidenced by the findings, healthcare providers have a critical role to play: highlighting how important is proper nutrition and mental health for the mother and her fetus; and screening, assessing, managing and linking pregnant women with suitable specialized care.

## Supporting information

S1 Table Correlations among the different variables. (XLSX)

S1 Supplementary Material English and Arabic Questionnaires. (DOCX)

## Abbreviations

BDS: Beirut Distress Scale
BMI: Body Mass Index
DEAPS: Disordered Eating Attitudes in Pregnancy Scale
FI: Food Insecurity
HFIAS: Household Food Insecurity Access Scale
IRB: Institutional Review Board
LMICs: Low- and Middle-income Countries
MD: Mediterranean Diet
MEDAS: Mediterranean Diet Adherence Screener
PHQ: Patient Health Questionnaire
PSQi: Pittsburgh Sleep Quality Index
PTSD: Posttraumatic Stress Disorder.
