# Supplementary material for: Association between food insecurity and mental health outcomes among a convenient sample of Lebanese pregnant women
Source: PLoS One. 2025 Sep 29;20(9):e0332581. doi: 10.1371/journal.pone.0332581 (PMC12478941; doi:10.1371/journal.pone.0332581)
Supplement: S1 Supplementary Material — (DOCX) [file pone.0332581.s002.docx]

# Supporting information

**Table S1.** Correlations among the different variables

| **Variable** | | **A_DEAPS** | | | | **P-value** | **PHQ-4 Anxiety** | | | | **P-value** | **PHQ-4 Depression** | | | | **P-value** | **BDS** | | | | **P-value** | **PSQI** | | | | **P-value** |
| --- | --- | --- | --- | --- | --- | --- | --- | --- | --- | --- | --- | --- | --- | --- | --- | --- | --- | --- | --- | --- | --- | --- | --- | --- | --- | --- |
|  |  | **No disordered eating** | | **Disordered eating** | |  | **No anxiety** | | **Anxiety** | |  | **No Depression** | | **Depression** | |  | **Moderate Distress** | | **High Distress** | |  | **Poor Sleep** | | **Good Sleep** | |  |
|  |  | **N** | **%** | **N** | **%** |  | **N** | **%** | **N** | **%** |  | **N** | **%** | **N** | **%** |  | **N** | **%** | **N** | **%** |  | **N** | **%** | **N** | **%** |  |
| **Pre-pregnancy BMI classification** | Normal | 63 | 48.50% | 8 | 57.10% | 0.865 | 36 | 50.70% | 35 | 47.90% | 0.125 | 40 | 51.30% | 31 | 47.00% | 0.423 | 10 | 41.70% | 61 | 50.80% | 0.189 | 35 | 42.70% | 36 | 58.10% | 0.059 |
|  | Underweight | 6 | 4.60% | 1 | 7.10% |  | 1 | 1.40% | 6 | 8.20% |  | 2 | 2.60% | 5 | 7.60% |  | 0 | 0.00% | 7 | 5.80% |  | 7 | 8.50% | 0 | 0.00% |  |
|  | Overweight | 34 | 26.20% | 3 | 21.40% |  | 22 | 31.00% | 15 | 20.50% |  | 22 | 28.20% | 15 | 22.70% |  | 10 | 41.70% | 27 | 22.50% |  | 23 | 28.00% | 14 | 22.60% |  |
|  | Obesity | 27 | 20.80% | 2 | 14.30% |  | 12 | 16.90% | 17 | 23.30% |  | 14 | 17.90% | 15 | 22.70% |  | 4 | 16.70% | 25 | 20.80% |  | 17 | 20.70% | 12 | 19.40% |  |
| **Pregnancy trimester** | First Trimester | 28 | 21.40% | 4 | 28.60% | **0.03** | 17 | 23.90% | 15 | 20.30% | 0.864 | 19 | 24.10% | 13 | 19.70% | 0.383 | 8 | 33.30% | 24 | 19.80% | 0.346 | 18 | 21.40% | 14 | 23.00% | 0.195 |
|  | Second Trimester | 47 | 35.90% | 9 | 64.30% |  | 27 | 38.00% | 29 | 39.20% |  | 33 | 41.80% | 23 | 34.80% |  | 8 | 33.30% | 48 | 39.70% |  | 28 | 33.30% | 28 | 45.90% |  |
|  | Third Trimester | 56 | 42.70% | 1 | 7.10% |  | 27 | 38.00% | 30 | 40.50% |  | 27 | 34.20% | 30 | 45.50% |  | 8 | 33.30% | 49 | 40.50% |  | 38 | 45.20% | 19 | 31.10% |  |
| **Parity** | No | 78 | 59.10% | 6 | 42.90% | 0.243 | 33 | 45.80% | 51 | 68.90% | **0.005** | 40 | 50.00% | 44 | 66.70% | **0.043** | 12 | 50.00% | 72 | 59.00% | 0.414 | 54 | 64.30% | 30 | 48.40% | 0.055 |
|  | Yes | 54 | 40.90% | 8 | 57.10% |  | 39 | 54.20% | 23 | 31.10% |  | 40 | 50.00% | 22 | 33.30% |  | 12 | 50.00% | 50 | 41.00% |  | 30 | 35.70% | 32 | 51.60% |  |
| **Sex of the fetus** | Male | 40 | 30.30% | 5 | 35.70% | 0.432 | 22 | 30.60% | 23 | 31.10% | 0.786 | 26 | 32.50% | 19 | 28.80% | 0.132 | 7 | 29.20% | 38 | 31.10% | 0.246 | 30 | 35.70% | 15 | 24.20% | 0.297 |
|  | Female | 51 | 38.60% | 3 | 21.40% |  | 25 | 34.70% | 29 | 39.20% |  | 24 | 30.00% | 30 | 45.50% |  | 6 | 25.00% | 48 | 39.30% |  | 30 | 35.70% | 24 | 38.70% |  |
|  | Don't Know | 41 | 31.10% | 6 | 42.90% |  | 25 | 34.70% | 22 | 29.70% |  | 30 | 37.50% | 17 | 25.80% |  | 11 | 45.80% | 36 | 29.50% |  | 24 | 28.60% | 23 | 37.10% |  |
| **Number of fetuses** | One | 124 | 93.90% | 14 | 100.00% | 0.343 | 69 | 95.80% | 69 | 93.20% | 0.492 | 75 | 93.80% | 63 | 95.50% | 0.652 | 23 | 95.80% | 115 | 94.30% | 0.757 | 80 | 95.20% | 58 | 93.50% | 0.657 |
|  | More than one | 8 | 6.10% | 0 | 0.00% |  | 3 | 4.20% | 5 | 6.80% |  | 5 | 6.20% | 3 | 4.50% |  | 1 | 4.20% | 7 | 5.70% |  | 4 | 4.80% | 4 | 6.50% |  |
| **Presence of constipation** | No | 70 | 53.00% | 9 | 64.30% | 0.422 | 41 | 56.90% | 38 | 51.40% | 0.498 | 47 | 58.80% | 32 | 48.50% | 0.215 | 19 | 79.20% | 60 | 49.20% | **0.007** | 38 | 45.20% | 41 | 66.10% | **0.012** |
|  | Yes | 62 | 47.00% | 5 | 35.70% |  | 31 | 43.10% | 36 | 48.60% |  | 33 | 41.20% | 34 | 51.50% |  | 5 | 20.80% | 62 | 50.80% |  | 46 | 54.80% | 21 | 33.90% |  |
| **Presence of anemia** | No | 87 | 65.90% | 12 | 85.70% | 0.132 | 55 | 76.40% | 44 | 59.50% | **0.029** | 63 | 78.80% | 36 | 54.50% | **0.002** | 22 | 91.70% | 77 | 63.10% | **0.006** | 50 | 59.50% | 49 | 79.00% | **0.013** |
|  | Yes | 45 | 34.10% | 2 | 14.30% |  | 17 | 23.60% | 30 | 40.50% |  | 17 | 21.20% | 30 | 45.50% |  | 2 | 8.30% | 45 | 36.90% |  | 34 | 40.50% | 13 | 21.00% |  |
| **Presence of hypertension** | No | 125 | 94.70% | 13 | 92.90% | 0.774 | 71 | 98.60% | 67 | 90.50% | **0.032** | 79 | 98.80% | 59 | 89.40% | **0.013** | 24 | 100.00% | 114 | 93.40% | 0.197 | 78 | 92.90% | 60 | 96.80% | 0.304 |
|  | Yes | 7 | 5.30% | 1 | 7.10% |  | 1 | 1.40% | 7 | 9.50% |  | 1 | 1.20% | 7 | 10.60% |  | 0 | 0.00% | 8 | 6.60% |  | 6 | 7.10% | 2 | 3.20% |  |
| **Presence of gestational diabetes** | No | 59 | 44.70% | 4 | 28.60% | 0.511 | 28 | 38.90% | 35 | 47.30% | 0.147 | 34 | 42.50% | 29 | 43.90% | 0.555 | 12 | 50.00% | 51 | 41.80% | 0.387 | 43 | 51.20% | 20 | 32.30% | 0.074 |
|  | Yes | 7 | 5.30% | 1 | 7.10% |  | 2 | 2.80% | 6 | 8.10% |  | 3 | 3.80% | 5 | 7.60% |  | 0 | 0.00% | 8 | 6.60% |  | 4 | 4.80% | 4 | 6.50% |  |
|  | Not Tested | 66 | 50.00% | 9 | 64.30% |  | 42 | 58.30% | 33 | 44.60% |  | 43 | 53.80% | 32 | 48.50% |  | 12 | 50.00% | 63 | 51.60% |  | 37 | 44.00% | 38 | 61.30% |  |
| **14-MEDAS** | Weak Adherence | 50 | 37.90% | 5 | 35.70% | 0.072 | 29 | 40.30% | 26 | 35.10% | 0.565 | 28 | 35.00% | 27 | 40.90% | 0.066 | 10 | 41.70% | 45 | 36.90% | 0.076 | 35 | 41.70% | 20 | 32.30% | 0.493 |
|  | Moderate to Fair Adherence | 75 | 56.80% | 6 | 42.90% |  | 37 | 51.40% | 44 | 59.50% |  | 43 | 53.80% | 38 | 57.60% |  | 10 | 41.70% | 71 | 58.20% |  | 44 | 52.40% | 37 | 59.70% |  |
|  | Good to Very Good Adherence | 7 | 5.30% | 3 | 21.40% |  | 6 | 8.30% | 4 | 5.40% |  | 9 | 11.20% | 1 | 1.50% |  | 4 | 16.70% | 6 | 4.90% |  | 5 | 6.00% | 5 | 8.10% |  |
| **Age category** | <= 30 years | 70 | 53.40% | 9 | 64.30% | 0.438 | 42 | 58.30% | 37 | 50.70% | 0.355 | 49 | 61.20% | 30 | 46.20% | 0.069 | 12 | 50.00% | 67 | 55.40% | 0.629 | 43 | 51.80% | 36 | 58.10% | 0.454 |
|  | > 30 years | 61 | 46.60% | 5 | 35.70% |  | 30 | 41.70% | 36 | 49.30% |  | 31 | 38.80% | 35 | 53.80% |  | 12 | 50.00% | 54 | 44.60% |  | 40 | 48.20% | 26 | 41.90% |  |
| **Residency** | Beirut/Mount Lebanon | 63 | 47.70% | 5 | 35.70% | 0.392 | 37 | 51.40% | 31 | 41.90% | 0.25 | 41 | 51.20% | 27 | 40.90% | 0.213 | 12 | 50.00% | 56 | 45.90% | 0.713 | 40 | 47.60% | 28 | 45.20% | 0.769 |
|  | Others | 69 | 52.30% | 9 | 64.30% |  | 35 | 48.60% | 43 | 58.10% |  | 39 | 48.80% | 39 | 59.10% |  | 12 | 50.00% | 66 | 54.10% |  | 44 | 52.40% | 34 | 54.80% |  |
| **Setting** | Rural | 65 | 49.20% | 7 | 50.00% | 0.957 | 33 | 45.80% | 39 | 52.70% | 0.407 | 31 | 38.80% | 41 | 62.10% | **0.005** | 5 | 20.80% | 67 | 54.90% | **0.002** | 42 | 50.00% | 30 | 48.40% | 0.847 |
|  | Urban | 67 | 50.80% | 7 | 50.00% |  | 39 | 54.20% | 35 | 47.30% |  | 49 | 61.20% | 25 | 37.90% |  | 19 | 79.20% | 55 | 45.10% |  | 42 | 50.00% | 32 | 51.60% |  |
| **Smoking** | No | 110 | 83.30% | 11 | 78.60% | 0.653 | 60 | 83.30% | 61 | 82.40% | 0.885 | 68 | 85.00% | 53 | 80.30% | 0.453 | 24 | 100.00% | 97 | 79.50% | **0.015** | 67 | 79.80% | 54 | 87.10% | 0.245 |
|  | Yes | 22 | 16.70% | 3 | 21.40% |  | 12 | 16.70% | 13 | 17.60% |  | 12 | 15.00% | 13 | 19.70% |  | 0 | 0.00% | 25 | 20.50% |  | 17 | 20.20% | 8 | 12.90% |  |
| **Employment status** | Unemployed | 76 | 57.60% | 9 | 64.30% | 0.628 | 33 | 45.80% | 52 | 70.30% | **0.003** | 40 | 50.00% | 45 | 68.20% | **0.027** | 9 | 37.50% | 76 | 62.30% | **0.024** | 51 | 60.70% | 34 | 54.80% | 0.477 |
|  | Employed | 56 | 42.40% | 5 | 35.70% |  | 39 | 54.20% | 22 | 29.70% |  | 40 | 50.00% | 21 | 31.80% |  | 15 | 62.50% | 46 | 37.70% |  | 33 | 39.30% | 28 | 45.20% |  |
| **Education level** | Intermediate/Secondary | 50 | 37.90% | 5 | 35.70% | 0.874 | 18 | 25.00% | 37 | 50.00% | **0.002** | 25 | 31.20% | 30 | 45.50% | 0.078 | 7 | 29.20% | 48 | 39.30% | 0.347 | 39 | 46.40% | 16 | 25.80% | **0.011** |
|  | University/equivalent | 82 | 62.10% | 9 | 64.30% |  | 54 | 75.00% | 37 | 50.00% |  | 55 | 68.80% | 36 | 54.50% |  | 17 | 70.80% | 74 | 60.70% |  | 45 | 53.60% | 46 | 74.20% |  |
| **Crowding index** | Not Crowded | 86 | 65.20% | 13 | 92.90% | **0.035** | 57 | 79.20% | 42 | 56.80% | **0.004** | 61 | 76.20% | 38 | 57.60% | **0.016** | 17 | 70.80% | 82 | 67.20% | 0.729 | 51 | 60.70% | 48 | 77.40% | **0.033** |
|  | Crowded | 46 | 34.80% | 1 | 7.10% |  | 15 | 20.80% | 32 | 43.20% |  | 19 | 23.80% | 28 | 42.40% |  | 7 | 29.20% | 40 | 32.80% |  | 33 | 39.30% | 14 | 22.60% |  |
| **Financial independency** | No | 59 | 44.70% | 5 | 35.70% | 0.696 | 24 | 33.30% | 40 | 54.10% | **<0.001** | 25 | 31.20% | 39 | 59.10% | **<0.001** | 7 | 29.20% | 57 | 46.70% | 0.233 | 45 | 53.60% | 19 | 30.60% | **0.014** |
|  | Yes | 42 | 31.80% | 6 | 42.90% |  | 35 | 48.60% | 13 | 17.60% |  | 37 | 46.20% | 11 | 16.70% |  | 11 | 45.80% | 37 | 30.30% |  | 25 | 29.80% | 23 | 37.10% |  |
|  | Prefer Not to Say | 31 | 23.50% | 3 | 21.40% |  | 13 | 18.10% | 21 | 28.40% |  | 18 | 22.50% | 16 | 24.20% |  | 6 | 25.00% | 28 | 23.00% |  | 14 | 16.70% | 20 | 32.30% |  |

**Supplementary Material 1:** English and Arabic Questionnaires

# Questionnaire (English)

*Consent to participate in a Survey*

*Food insecurity among a convenient sample of pregnant women*

*I would like to invite you to participate in a research project by completing the following survey. We are a group of researchers at the Lebanese American University. The purpose of this survey is to explore the issue of food insecurity among pregnant women, that is, not having sufficient food in terms of quantity, quality, and diversity to ensure a healthy and active life. This study also explores the association between food insecurity, and sociodemographic and pregnancy characteristics, as well as its association with lifestyle, diet quality, and health outcomes.*

*There are no known risks, harms or discomforts associated with this study beyond those encountered in normal daily life. The information you provide will be used to enhance our understanding on the matter to improve the lifestyle of pregnant women. You will not directly benefit from participation in this study. The study will involve 200 participants. Completing the survey will take 15 to 20 minutes of your time.*

*By continuing with the survey, you agree with the following statements:*

1. *I have been given sufficient information about this research project.*
2. *I understand that my answers will not be released to anyone and my identity will remain anonymous. My name will not be written on the questionnaire nor be kept in any other records.*
3. **When the results of the study are reported, I will not be identified by name or any other information that could be used to infer my identity.** *Only researchers will have access to view any data collected during this research however data cannot be linked to me.*
4. *I understand that I may withdraw from this research any time I wish and that I have the right to skip any question I don’t want to answer.*
5. *I understand that my refusal to participate will not result in any penalty or loss of benefits to which I otherwise am entitled to.*
6. *I have been informed that the research abides by all commonly acknowledged ethical codes and that the research project has been reviewed and approved by the Institutional Review Board at the Lebanese American University*
7. *I understand that if I have any additional questions, I can ask the research team listed below.*
8. *I have read and understood all statements on this form.*
9. *I voluntarily agree to take part in this research project by completing the following survey.*

**This study has been reviewed and approved by the LAU IRB: LAU.SAS.RR2.20/Jan/2023**

| Yes - I agree to participate in this online survey. |
| --- |
| No - Exit survey |

**Food Insecurity Scale**

1. **The Household Food Insecurity Access Scale (HFIAS)**

| 1. In the past four weeks, did you worry that your household would not have enough food?   **1a. How often did this happen?**  1 = Rarely (once or twice in the past four weeks)  2 = Sometimes (three to ten times in the past four weeks)  3 = Often (more than ten times in the past four weeks) | **0 = No (skip to Q2)**  **1 = Yes** |
| --- | --- |
| 1. In the past four weeks, were you or any household member not able to eat the kinds of foods you preferred because of a lack of resources?   **2a. How often did this happen?**  1 = Rarely (once or twice in the past four weeks)  2 = Sometimes (three to ten times in the past four weeks)  3 = Often (more than ten times in the past four weeks) | **0 = No (skip to Q3)**  **1 = Yes** |
| 1. In the past four weeks, did you or any household member have to eat a limited variety of foods due to a lack of resources?   **3a.** **How often did this happen?**  1 = Rarely (once or twice in the past four weeks)  2 = Sometimes (three to ten times in the past four weeks)  3 = Often (more than ten times in the past four weeks) | **0 = No (skip to Q4)**  **1 = Yes** |
| 1. In the past four weeks, did you or any household member have to eat some foods that you really did not want to eat because of a lack of resources to obtain other types of food?   **4a.** **How often did this happen?**  1 = Rarely (once or twice in the past four weeks)  2 = Sometimes (three to ten times in the past four weeks)  3 = Often (more than ten times in the past four weeks) | **0 = No (skip to Q5)**  **1 = Yes** |
| 1. In the past four weeks, did you or any household member have to eat a smaller meal than you felt you needed because there was not enough food?   **5a.** **How often did this happen?**  1 = Rarely (once or twice in the past four weeks)  2 = Sometimes (three to ten times in the past four weeks)  3 = Often (more than ten times in the past four weeks) | **0 = No (skip to Q6)**  **1 = Yes** |
| 1. In the past four weeks, did you or any other household member have to eat fewer meals in a day because there was not enough food?   **6a.** **How often did this happen?**  1 = Rarely (once or twice in the past four weeks)  2 = Sometimes (three to ten times in the past four weeks)  3 = Often (more than ten times in the past four weeks) | **0 = No (skip to Q7)**  **1 = Yes** |
| 1. In the past four weeks, was there ever no food to eat of any kind in your household because of lack of resources to get food?   **7a.** **How often did this happen?**  1 = Rarely (once or twice in the past four weeks)  2 = Sometimes (three to ten times in the past four weeks)  3 = Often (more than ten times in the past four weeks) | **0 = No (skip to Q8)**  **1 = Yes** |
| 1. In the past four weeks, did you or any household member go to sleep at night hungry because there was not enough food?   **8a.** **How often did this happen?**  1 = Rarely (once or twice in the past four weeks)  2 = Sometimes (three to ten times in the past four weeks)  3 = Often (more than ten times in the past four weeks) | **0 = No (skip to Q9)**  **1 = Yes** |
| 1. In the past four weeks, did you or any household member go a whole day and night without eating anything because there was not enough food?   **9a.** **How often did this happen?**  1 = Rarely (once or twice in the past four weeks)  2 = Sometimes (three to ten times in the past four weeks)  3 = Often (more than ten times in the past four weeks) | **0 = No (questionnaire is finished)**  **1 = Yes** |

**Eating-Related Questions and Scales**

1. **The Rapid Eating Assessment for Participants – Shortened Version (REAP-S)**

| **For the previous week, how often did you:** | **Usually Often**  **(1)** | **Sometimes**  **(2)** | | **Rarely/ Never**  **(3)** | **Does not apply to me (0)** |
| --- | --- | --- | --- | --- | --- |
| 1. Skip breakfast? |  |  | |  |  |
| 1. Eat 4 or more meals from sit-down or take out restaurants? |  |  | |  |  |
| 1. Eat less than 2 servings of whole grain products or high-fiber starches a day?   *Serving = 1 slice of whole wheat bread, 1 cup whole grain cereal like shredded wheat, high-fiber cereals, oatmeal, bran cereals, 3-4 whole grain crackers, ½ cup brown rice or whole wheat pasta or burghul, 1 small boiled or baked potatoes* |  |  | |  |  |
| 1. Eat less than 2 servings of fruit a day?   *Serving = 1 medium fruit (1 medium apple or 1 medium banana or 2 small apricots or 2 small kiwi or 2 small plums…) or ¾ cup 100% fresh fruit juice* |  |  | |  |  |
| 1. Eat less than 2 servings of vegetables a day?   *Serving = ½ cup vegetables or 1 cup leafy raw vegetables* |  |  | |  |  |
| 1. Eat or drink less than 2 servings of milk, yogurt, or cheese a day?   *Serving = 1 cup milk or yogurt; 45-60 g cheese* |  |  | |  |  |
| 1. Eat more than 8 servings (see sizes below) of meat, chicken, turkey or fish per day?   *Note: 3 servings of meat or chicken is the size of a deck of cards or ONE of the following: 1 regular hamburger, 1 chicken breast or leg (thigh and drumstick)* |  |  | |  | Rarely eat meat, chicken, turkey or fish ☐ |
| 1. Use regular processed meats (like bologna, salami, hotdogs, soujouk, makanek) instead of low-fat processed meats (like roast beef, turkey, lean ham; low-fat cold cuts/hotdogs)? |  |  | |  | Rarely eat processed meats ☐ |
| 1. Eat fried foods such as fried chicken, fried fish, French fries, fried falafel |  |  | |  |  |
| 1. Eat regular potato chips, crackers, regular popcorn, nuts instead of pretzels, low-fat chips or low-fat crackers, air-popped popcorn? |  |  | |  | Rarely eat these snack foods ☐ |
| 1. Add butter, margarine or oil to bread, potatoes, rice or vegetables at the table? |  |  | |  |  |
| 1. Eat sweets like cake, cookies, pastries, donuts, muffins, chocolate, candies…, or Arabic sweets like baklava, kunafa… more than 2 times per day |  |  | |  |  |
| 1. Drink 500 ml (2 cups) or more of non-diet soda, fruit drink/punch a day?   *Note: 1 regular can of soda = 330 ml* |  |  | |  |  |
|  | **Yes** | | | **No** | |
| 1. You or a member of your family usually shops and cooks rather than eating sit-down or take-out restaurant food? |  | | | |  |
| 1. Usually feel well enough to shop or cook |  | | | |  |
| 1. How willing are you to make changes in your eating habits in order to be healthier? | **1**  **Very willing** | **2** | **3** | **4** | **5**  **Not at all willing** |
|  |  |  |  |  |  |

1. **Mediterranean Diet Adherence Screener (MEDAS)**

| **Please answer each of the following questions by choosing the option that best applies to you** | | |
| --- | --- | --- |
|  | **No** | **Yes** |
| 1. Do you use olive oil as the principal source of fat for cooking? | 0 | 1 |
| 1. How much olive oil do you consume per day (including that used in frying, salads, meals eaten away from home, etc.)? | ____ Tablespoons | |
| 1. How many servings of vegetables do you consume per day? Count garnish and side servings as 1/2 point; a full serving is 200 g. | ____ Servings | |
| 1. How many pieces of fruit (including fresh-squeezed juice) do you consume per day? | ____ Servings | |
| 1. How many servings of red meat, hamburger, or sausages do you consume per day? A full serving is 100–150 g. | ____ Servings | |
| 1. How many servings (12 g) of butter, margarine, or cream do you consume per day? | ____ Servings | |
| 1. How many carbonated and/or sugar-sweetened beverages do you consume per day? | ____ Glasses | |
| 1. Do you drink wine? How much do you consume per week? | ____ Glasses | |
| 1. How many servings (150 g) of pulses (beans, lentils, and peas) do you consume per week? | ____ Servings | |
| 1. How many servings of fish/seafood do you consume per week? (100–150 g of fish, 4–5 pieces or 200 g of seafood) | ____ Servings | |
| 1. How many times do you consume commercial (not homemade) pastry such as cookies or cake per week? | ____ Times | |
| 1. How many times do you consume nuts per week? (1 serving = 30 g) | ____ Servings | |
| 1. Do you prefer to eat chicken, turkey or rabbit instead of beef, pork, hamburgers, or sausages? | 0 | 1 |
| 1. How many times per week do you consume boiled vegetables, pasta, rice, or other dishes with a sauce of tomato, garlic, onion, or leeks sautéed in olive oil? | ____ Times | |

1. **Disordered Eating Attitudes in Pregnancy Scale (A-DEAPS)**

| **At any time during pregnancy:** | Yes | No |
| --- | --- | --- |
| 1. I have felt distressed about the changes to my body and/or eating habits during pregnancy. |  |  |
| 2. I have attempted to stop the changes occurring to my body during pregnancy. |  |  |
| 3. I have felt anxious about eating in general, or about eating certain foods. |  |  |
| 4. I have felt distressed after eating because of its effect on my weight and shape. |  |  |
| 5. I have noticed that what I allow myself to eat and how much I can eat is connected to rules and conditions. |  |  |
| 7. I worried that I have, or will, become ‘fat’ during pregnancy. |  |  |
| 9. I have spent considerable time researching the most effective ways to minimize how much weight I gain while pregnant. |  |  |
| 10. I have spent considerable time researching how I can rapidly lose weight after I have given birth. |  |  |
| 13. I have wanted my pregnancy body to be small, like I am “just bump” (i.e., only my stomach appears to have grown, with no weight or shape changes to other areas of my body). |  |  |
| 14. I have found myself frequently (at least once a week) comparing my weight, shape, size, or eating habits to other women. |  |  |

**Mental Health-Related Questions**

1. **PHQ-4**

| **Over the last 2 weeks, how often have you   been bothered by the following problems?** | Not  at all | Several days | More than half the days | Nearly every day |
| --- | --- | --- | --- | --- |
| 1. Feeling nervous, anxious or on edge | 0 | 1 | 2 | 3 |
| 2. Not being able to stop or control worrying | 0 | 1 | 2 | 3 |
| 3. Little interest or pleasure in doing things | 0 | 1 | 2 | 3 |
| 4. Feeling down, depressed, or hopeless | 0 | 1 | 2 | 3 |

1. **The Pittsburgh Sleep Quality Index (PSQI)**

| **The following questions relate to your usual sleep habits during the past month only. Your answers should indicate the most accurate reply for the majority of days and nights in the past month. Please answer all questions.** | | | | |
| --- | --- | --- | --- | --- |
| 1. During the past month, what time have you usually gone to bed at night? ______________________ | | | | |
| 1. During the past month, how long (in minutes) has it usually taken you to fall asleep each night? _______________________ | | | | |
| 1. During the past month, what time have you usually gotten up in the morning? _________________ | | | | |
| 1. During the past month, how many hours of actual sleep did you get at night? (This may be different than the number of hours you spent in bed.) ___________________________ | | | | |
|  | **Not during the past month** | **Less than once a week** | **Once or twice a week** | **Three or more times a week** |
| 1. During the past month, how often have you had trouble sleeping because you   a. Cannot get to sleep within 30 minutes  b. Wake up in the middle of the night or early morning  c. Have to get up to use the bathroom  d. Cannot breathe comfortably  e. Cough or snore loudly  f. Feel too cold  g. Feel too hot  h. Have bad dreams  i. Have pain  j. Other reason(s), please describe, including how often you have had trouble sleeping because of this reason (s): |  |  |  |  |
| 1. During the past month, how often have you taken medicine to help you sleep (prescribed or “over the counter”)? |  |  |  |  |
| 1. During the past month, how often have you had trouble staying awake while driving, eating meals, or engaging in social activity? |  |  |  |  |
|  | **No problem at all** | **Only a very slight problem** | **Somewhat of a problem** | **A very big problem** |
| 1. During the past month, how much of a problem has it been for you to keep up enthusiasm to get things done? |  |  |  |  |
|  | **Very good** | **Fairly good** | **Fairly bad** | **Very bad** |
| 1. During the past month, how would you rate your sleep quality overall? |  |  |  |  |
|  | **No bed**  **partner or**  **room mate** | **Partner/ roommate in other room** | **Partner in**  **same room but not same bed** | **Partner in same bed** |

1. **The Beirut Distress Scale (BDS-10)**

| **During the past month:** | **Never**  **(0)** | **Rarely**  **(1)** | **Moderately**  **(2)** | **Very much**  **(3)** |
| --- | --- | --- | --- | --- |
| 1. My mood changes rapidly for tiny matters |  |  |  |  |
| 1. I get angry for ridiculous reasons |  |  |  |  |
| 1. My ideas are puzzled |  |  |  |  |
| 1. I isolate myself |  |  |  |  |
| 1. I have difficulty concentrating |  |  |  |  |
| 1. I find it difficult to relax |  |  |  |  |
| 1. I have memory troubles |  |  |  |  |
| 1. I have constipation or diarrhea |  |  |  |  |
| 1. I have stomach cramps |  |  |  |  |
| 1. I have stomach heartburn |  |  |  |  |

**Pregnancy Characteristics**

1. **How far along is your pregnancy is in terms of weeks or months? ­­­­­­­­­**______________ weeks **or** ­­­­__________ months
2. **Is this your first pregnancy?** No Yes
3. **If this is not your first pregnancy, please state the number of prior pregnancies”** __________
4. **How many fetuses are you carrying?** 1 2 3 or more
5. **What is the gender of the fetus?** Male Female Does not know
6. **Do you have any pregnancy complications? [check all that apply]**

None Constipation Anemia Hypertension Other, specify: ___________

1. **Did you develop diabetes during this pregnancy?**  Tested, Yes Tested, No Not tested yet
2. **What is your height?** ___________
3. **What was your weight before pregnancy?** _______________
4. **What is your current weight?** _______________
5. **Do you take any supplements? [check all that apply]**

No Yes Iron Calcium Vitamin B9 Iodine Multivitamin Other, specify

1. **In case you take supplements, who prescribed them? [check all that apply]**

No one (self)  A family member/friend  My health care provider (physician, midwife)  The pharmacist

1. **Who is your caregiver?** Private physician Physician, PHC Midwife Other, specify: ___________
2. **Do you intend to breastfeed?** Yes, exclusive Yes, mixed No Did not decide yet

**Sociodemographic Characteristics**

1. **What is your age?**
2. **Where do you live?** Beirut Mount Lebanon North Bekaa South
3. **Do you live in a rural or urban area?** Urban Rural
4. **What is your educational level?** Illiterate Primary Intermediate Secondary/equivalent University/equivalent
5. **What is your marital status?**

Single Married In a solid relationship, unmarried

Widowed Divorced

1. **What is your employment status?**

Not employed Full-time employee, specify: ______ Part-time employee, specify: ______

1. **Who is your insurer? [check all that apply]**

None Private insurance NSSF Coop Army Other, specify: ______

1. **Do you currently smoke cigarettes?** No Yes, specify: ___________ cigarettes/day
2. **Do you currently smoke waterpipe?** No Yes
3. **If you currently smoke waterpipe, how often?**  ≤1/day: 10-30 min/few puffs >1/day, specify number of minutes: ______
4. **Are you exposed to passive smoking, that is the involuntary inhaling of smoke from other people's cigarettes, waterpipe…?** No Yes

**Financial Characteristics**

1. **How many persons live in the house, including you?** _______
2. **What is the number of rooms in the house, excluding kitchen and bathrooms?** ________
3. **What is your family income per month in $?** ________
4. **Are you financially independent?** No Yes ☐Prefer not to say

**Questionnaire (Arabic)**

**انعدام الأمن الغذائي عند عينة من النساء الحوامل**

أود أن أدعوكم للمشاركة في مشروع بحثي من خلال استكمال الاستبيان التالي. نحن مجموعة من الباحثين في الجامعة اللبنانية الأمريكيةׅ الغرض من هذا الاستبيان هو دراسةانعدام الأمن الغذائي بين النساء الحوامل ، أي عدم وجود غذاء كافٍ من حيث الكمية والنوعية والتنوع لضمان حياة صحية ونشيطة. تستكشف هذه الدراسة أيضًا العلاقة بين انعدام الأمن الغذائي والخصائص الاجتماعية والديموغرافية والحمل فضلاً عن ارتباطها بنمط الحياة وجودة النظام الغذائي والنتائج الصحيةׅ

لا توجد مخاطر أو أضرار أو مضايقات معروفة مرتبطة بهذه الدراسة بخلاف تلك التي نواجهها في الحياة اليومية العادية. سيتم استخدام المعلومات التي تقدمها لتعزيز فهمنا لهذه المسألة لتحسين نمط حياة النساء الحوامل. لن تستفيد بشكل مباشر من المشاركة في هذه الدراسة. ستشمل الدراسة 200 مشارك. سيستغرق إكمال الاستبيان من 15 إلى 20 دقيقة من وقتكׅ

من خلال الاستمرار في الاستبيان ، فإنك توافق مع العبارات التالية :

1. لقد أعطيت ما يكفي من المعلومات حول هذا المشروع البحثي .
2. لن يتم التصريح او الافراج عن اجاباتي الى اي شخص ، وسوف تبقى هويتي مجهولة. لن يكتب اسمي على الاستبيان ولن يحفظ في أي سجلات أخرى.
3. . **عندما يتم تقديم نتائج الدراسة ، لن يتم التعرف الي بالاسم أو أي معلومات أخرى يمكن أن تستخدم للاستدلال الى هويت**ي**.** الباحثون فقط لديهم الحق في الاستطلاع على البيانات التي تم جمعها خلال هذا البحث لكن البيانات لا يمكن أن تكون مرتبطة بي.
4. ادرك ان مشاركتي طوعية وبإمكاني الإنسحاب من هذا البحث في أي وقت اشاء أو تخطي أي سؤال لا اشعر بالرد عليه.
5. انا ادرك بان رفضي للمشاركة لا ينتج عنه أي جزاء أو فقدان أي من الحقوق التي أنا مؤهل لها.
6. لقد تم ابلاغي ان البحث يلتزم بجميع القوانين الأخلاقية المعترف بها, وبان هذه الدراسة تمت مراجعتها والموافقة عليها من قبل مكتب اللجنة الأخلاقية في الجامعة اللبنانية الأميركية.
7. أنا أفهم أنه إذا كان لديي أي أسئلة إضافية يمكنني ان اطلب من فريق البحث المدرجة اسمائهم في القائمة ادناه.
8. لقد قرأت وفهمت كل البيانات الواردة في هذا النموذج
9. أنا أوافق طوعا للمشاركة في هذا المشروع البحثي من خلال استكمال الاستيبان التالي.

تم الموافقة على هذا الاستبيان من قبل مكتب اللجنة الأخلاقية في الجامعة اللبنانية الأميركية: **LAU.SAS.RR2.20/Jan/2023**

| نعم - أوافق على المشاركة في هذا الاستبيان عبر الإنترنت. |
| --- |
| لا - الخروج من الاستبيان |

1. **أداة قياس حالة الأمن الغذائي في المنزل**

| 0= لا (انتقل للسؤال 2)  1= نعم | 1. في الأسابيع الاربعة السابقة، هل قلقت بأن منزلك لا يحتوي على الطعام الكافي ؟  1- أ. كم مرة حدث ذلك ؟  1= نادرا (مرة أو مرتين في الأسابيع الأربعة السابقة)  2= أحيانا (3 إلى 10 مرات في الأسابيع الأربعة السابقة)  3= غالبا (أكثر من 10 مرات في الأسابيع الأربعة السابقة) |
| --- | --- |
| 0= لا (انتقل للسؤال 3)  1= نعم | 2. في الأسابيع الأربعة السابقة، هل أنت أو واحد من أفراد الأسرة لم يتمكن من تناول أنواع الأطعمة المفضلة لديه لعدم وجود الموارد الكافية ؟  2- أ. كم مرة حدث ذلك ؟  1= نادرا (مرة أو مرتين في الأسابيع الأربعة السابقة)  2= أحيانا (3 إلى 10 مرات في الأسابيع الأربعة السابقة)  3= غالبا (أكثر من 10 مرات في الأسابيع الأربعة السابقة) |
| 0= لا (انتقل للسؤال 4)  1= نعم | 3. في الأسابيع الأربعة السابقة، هل أنت أو أحد أفراد الأسرة لم يتمكن من تناول الأطعمة المفضلة لديه لعدم وجود الموارد الكافية ؟  3- أ. كم مرة حدث ذلك ؟  1= نادرا (مرة أو مرتين في الأسابيع الأربعة السابقة)  2= أحيانا (3 إلى 10 مرات في الأسابيع الأربعة السابقة)  3= غالبا (أكثر من 10 مرات في الأسابيع الأربعة السابقة) |
| 0= لا (انتقل للسؤال 5)  1= نعم | 4. في الأسابيع الأربعة السابقة، هل أنت أو واحد من أفراد الأسرة وجب عليه تناول نوع من الطعام لم يكن يريد تناوله لعدم وجود الموارد للحصول على أنواع أخرى من الطعام ؟  4- أ. كم مرة حدث ذلك ؟  1= نادرا (مرة أو مرتين في الأسابيع الأربعة السابقة)  2= أحيانا (3 إلى 10 مرات في الأسابيع الأربعة السابقة)  3= غالبا (أكثر من 10 مرات في الأسابيع الأربعة السابقة) |
| 0= لا (انتقل للسؤال 6)  1= نعم | 5. في الأسابيع الأربعة السابقة، هل أنت أو أحد من أفراد الأسرة وجب عليه تناول نوع من الطعام لم يكن يريد تناوله لعدم وجود الموارد للحصول على أنواع اخرى من الطعام ؟  5- أ. كم مرة حدث ذلك ؟  1= نادرا (مرة أو مرتين في الأسابيع الأربعة السابقة)  2= أحيانا (3 إلى 10 مرات في الأسابيع الأربعة السابقة)  3= غالبا (أكثر من 10 مرات في الأسابيع الأربعة السابقة) |
| 0= لا (انتقل للسؤال 7)  1= نعم | **6.** في الأسابيع الأربعة السابقة، هل أنت أو أحد أفراد الأسرة وجب عليه تناول أقل في اليوم لعدم وجود كمية كافية من الطعام ؟  6- أ. كم مرة حدث ذلك ؟  1= نادرا (مرة أو مرتين في الأسابيع الأربعة السابقة)  2= أحيانا (3 إلى 10 مرات في الأسابيع الأربعة السابقة)  3= غالبا (أكثر من 10 مرات في الأسابيع الأربعة السابقة) |
| 0= لا (انتقل للسؤال 8)  1= نعم | 7. في الأسابيع الأربعة السابقة، هل، في أي وقت، لم يتواجد أي نوع من الطعام في المنزل لعدم وجود الموارد للحصول على الطعام ؟  7- أ. كم مرة حدث ذلك ؟  1= نادرا (مرة أو مرتين في الأسابيع الأربعة السابقة)  2= أحيانا (3 إلى 10 مرات في الأسابيع الأربعة السابقة)  3= غالبا (أكثر من 10 مرات في الأسابيع الأربعة السابقة) |
| 0= لا (انتقل للسؤال 9)  1= نعم | 8. في الأسابيع الأربعة المقبلة، هل أنت أو أحد أفراد الأسرة ذهب إلى النوم في الليل جائعا لعدم توفر الطعام الكافي ؟  8- أ. كم مرة حدث ذلك ؟  1= نادرا (مرة أو مرتين في الأسابيع الأربعة السابقة)  2= أحيانا (3 إلى 10 مرات في الأسابيع الأربعة السابقة)  3= غالبا (أكثر من 10 مرات في الأسابيع الأربعة السابقة) |
| 0= لا (انتهى الاستبيان)  1= نعم | 9. في الأسابيع الأربعة السابقة، هل أنت أو أحد أفراد الأسرة بقي 24 ساعة دون تناول أي شيء لعدم توفر الطعام الكافي؟  9- أ. كم مرة حدث ذلك ؟  1= نادرا (مرة أو مرتين في الأسابيع الأربعة السابقة)  2= أحيانا (3 إلى 10 مرات في الأسابيع الأربعة السابقة)  3= غالبا (أكثر من 10 مرات في الأسابيع الأربعة السابقة) |
|  | |

1. **تقييم الأكل السريع لدى المشاركين – النسخة المختصرة**

| **في الأسبوع الماضي، ما معدّل قيامك بما يلي:** | **غالباً في العادة**  **(1)** | **أحياناً**  **(2)** | | **نادراً/ مطلقاً**  **(3)** | **لا ينطبق عليّ**  **(0)** |
| --- | --- | --- | --- | --- | --- |
| 1. عدم تناول وجبة الفطور؟ |  |  | |  |  |
| 1. تناول 4 وجبات أو أكثر في المطاعم سواء جلوساً أو من خلال الطلبات الخارجية؟ |  |  | |  |  |
| 1. تناول أقلّ من حصّتين من منتجات الحبوب الكاملة أو النشويات الغنية بالألياف في اليوم؟   *الحصّة = قطعة واحدة من خبز القمح الكامل، فنجان واحد من الحبوب الكاملة مثل حبوب القمح المبشورة، الحبوب عالية الألياف، دقيق الشوفان، حبوب النخالة، 3-4 مقرمشات الحبوب الكاملة، ½ كوب من الأرز البني أو المعكرونة من القمح الكامل أو برغل، 1 حبة بطاطس صغيرة مسلوقة أو مخبوزة.* |  |  | |  |  |
| 1. تناول أقلّ من حصتين من الفاكهة في اليوم؟   *الحصّة = حبة فاكهة واحدة ذات حجم متوسط (تفاحة متوسطة الحجم أو موزة متوسطة الحجم أو مشمشتان صغيرتان أو حبتان صغيرتان من الكيوي أو خوختان صغيرتان...) أو ¾ كوب من عصير الفاكهة* |  |  | |  |  |
| 1. تناول أقلّ من حصتين من الخضار في اليوم؟   *الحصّة = ½ كوب من الخضار أو فنجان واحد من الخضار النيّئة الورقية* |  |  | |  |  |
| 1. تناول أو شرب أقلّ من حصتين من الحليب، أو اللبن، أو الجبنة في اليوم؟   *الحصّة = فنجان من الحليب أو اللبن؛ 45-60 غ من الجبنة* |  |  | |  |  |
| 1. تناول أكثر من 8 حصص (راجع الأحجام أدناه) من اللحم، الدجاج، الحبش أو السمك في اليوم؟   *ملاحظة: 3 حصص من اللحم أو الدجاج تعادل حجم مجموعة ورق اللعب أو حصّة ممّا يلي: 1 هامبرغر حجم عادي، 1 صدر أو ساق دجاج (فخذ أو ربلة)* |  |  | |  | نادراً ما أتناول اللحم، الدجاج، الحبش أو السمك  ☐ |
| 1. استخدام اللحوم المصنّعة بطريقة عادية (مثل بولونيا، سلامي، هوت دوغ، سجق، مقانق) عوض اللحوم المصنّعة بدهون أقلّ (مثل الروستو، الحبش، لحم الهام الخالي من الدهون؛ الهوت دوغ/ اللحوم الباردة القليلة الدسم)؟ |  |  | |  | نادراً ما أتناول اللحوم المصنّعة ☐ |
| 1. تناول الأطعمة المقلية مثل الدجاج المقلي، السمك المقلي، البطاطس المقلية، الفلافل المقلية |  |  | |  |  |
| 1. تناول رقائق البطاطس العادية، المقرمشات، الفوشار العادي، المكسرات بدلاً من البريتزل، رقائق البطاطس القليلة الدهون أو المقرمشات القليلة الدهون، أو الفوشار المحضّر بتقنية الهواء الساخن؟ |  |  | |  | نادراً ما أتناول هذه الوجبات الخفيفة ☐ |
| 1. إضافة الزبدة، المارغرين أو الزيت مع الخبز، البطاطس، الأرز أو الخضار على المائدة؟ |  |  | |  |  |
| 1. تناول الحلويات مثل الكيك، والكوكيز، والمعجنات، والدوناتس، والمافن، والشوكولاتة، والسكاكر...، أو الحلويات العربية مثل البقلاوة، والكنافة أكثر من مرتين في اليوم |  |  | |  |  |
| 1. شرب 500 مل (كوبان أو أكثر من المشروبات الغازية العادية، مشروبات الفواكه أو الكوكتيل في اليوم؟   *ملاحظة: عبوة واحدة من المشروبات الغازية العادية = 330 مل* |  |  | |  |  |
|  | **نعم** | | | **كلا** | |
| 1. هل تتسوق أنت أو أحد أفراد أسرتك ومن ثم تطهو الطعام بدلاً من الجلوس في المطاعم أو طلب الوجبات الخارجية؟ |  | | | |  |
| 1. أشعر بالعادة بحسن الحال الكافي للتسوق أو الطهو |  | | | |  |
| 1. ما مدى استعدادك لإحداث تغييرات في عادات الأكل لتكون صحيةً أكثر؟ | **1**  **في أتمّ الاستعداد** | **2** | **3** | **4** | **5**  **غير مستعدّ على الإطلاق** |
|  |  |  |  |  |  |

1. **تقييم النظام الغذائي المتوسطي**

| **يرجى الإجابة على أفضل ما في ذاكرتك.**  **من فضلك لا تكتب أي شيء بجانب كلمة "نقاط".** | | |
| --- | --- | --- |
|  | **كلا** | **نعم** |
| 1. هل تستخدم زيت الزيتون كدهن رئيسي للطهي؟ | 0 | 1 |
| 1. ما مقدار زيت الزيتون الذي تستهلكه في يوم معين (بما في ذلك الزيت المستخدم للقلي و السلطات والوجبات خارج المنزل و ما إلى ذلك)؟ | ________ ملعقة طعام | |
| 1. كم عدد حصص الخضار التي تتناولها في اليوم؟ (حصة واحدة: 200 غرام [اعتبر الأطباق الجانبية نصف حصة]). | ________ حصة / حصص | |
| 1. كم عدد وحدات الفاكهة (بما في ذلك عصائر الفاكهة الطبيعية) التي تتناولها يوميا (وحدة فاكهة: 1 تفاحة متوسطة أو 1 موزة متوسطة أو 2 مشمش صغير أو 2 فاكهة كيوي صغيرة أو 2 برقوق صغير إلخ ... أو نصف كوب من عصير الفاكهة الطبيعي)؟ | ________ فاكهة / فواكه | |
| 1. كم عدد حصص اللحوم الحمراء أو الهامبرغر أو منتجات اللحوم (لحم الخنزير، السجق، إلخ) التي تتناولها في الأسبوع؟ (حصة واحدة: 100-150 غرام). | ________ حصة / حصص | |
| 1. كم عدد حصص الزبدة أو المارجرين أو الكريمة التي تتناولها في الأسبوع؟ (حصة واحدة: 12 غرام). | ________ حصة / حصص | |
| 1. كم عدد المشروبات الحلوة أو الغازية التي تشربها في الأسبوع؟ | ________ كوب / أكواب | |
| 1. كم تشرب من النبيذ في الأسبوع؟ | ________ كوب / أكواب | |
| 1. كم عدد حصص البقوليات (عدس، فاصوليا، فول وبازلاء...) التي تتناولها في الأسبوع؟ (حصة واحدة: 150 غرام). | ________ حصة / حصص | |
| 1. كم عدد حصص الأسماك أو المحار التي تتناولها في الأسبوع؟ (حصة واحدة من 100-150 غرام من الأسماك أو 4-5 وحدات أو 200 غرام من المحار). | ________ حصة / حصص | |
| 1. كم مرة في الأسبوع تتناول حلويات أو معجنات تجارية (غير مصنوعة في المنزل)، مثل الكعك و البسكوت و الكيك أو الكاسترد)؟ | ________ مرة | |
| 1. حصص المكسرات (بما في ذلك فستق عبيد) التي تتناولها في الأسبوع كم عدد ؟ (حصة واحدة 30 غرام). | ________ حصة / حصص | |
| 1. هل تفضل تناول لحم الدجاج أو الديك الرومي أو الأرنب بدلاً من لحم العجل أو لحم الخنزير أو الهمبرغر أو السجق؟ | 0  كلا | 1  نعم |
| 1. كم مرة في الأسبوع تتناول الخضار أو المعكرونة أو الأرز أو غيرها من الأطباق المتبلة بصلصة الطماطم (ممزوجة أو غير مخلوطة بالبصل ، الكراث ، الثوم أو زيت الزيتون)؟ | ____ مرة | |

1. **النسخة العربية من مقياس اضطراب الأكل في الحمل**

| لا | نعم | **في أي وقت أثناء الحمل** |
| --- | --- | --- |
|  |  | 1. لقد شعرت بالضيق بسبب التغييرات التي طرأت على جسدي و / أو على عادات أكلي أثناء الحمل |
|  |  | 2. لقد حاولت إيقاف التغييرات التي تحدث في جسدي أثناء الحمل |
|  |  | 3. لقد شعرت بالقلق بشأن تناول الطعام بشكل عام، أو بشأن تناول أطعمة معيّنة |
|  |  | 4. لقد شعرت بالضيق بعد الأكل لما له من تأثير على وزني وشكلي |
|  |  | 5. لقد لاحظت أنّ ما أسمح لنفسي بتناوله ومقدار ما يمكنني تناوله مرتبطَين بقواعد وشروط |
|  |  | 6. قلقت بشأن أنّني أصبحت أو سأصبح "سمينةً" أثناء الحمل |
|  |  | 7. لقد قضيت وقتًا طويلاً في البحث عن أكثر الطرق فعاليّة لتقليل مقدار الوزن الذي أكسبه أثناء الحمل |
|  |  | 8. لقد قضيت وقتًا طويلاً في البحث عن كيفية فقدان الوزن بسرعة بعد الولادة |
|  |  | 9. قد أردت أن يكون جسمي نحيفًا أثناء الحمل (أي أن يبدو أنّ بطني فقط قد نما، مع عدم تغيّر وزني وشكل مناطق أخرى من جسمي) |
|  |  | 10. لقد وجدت نفسي بشكل متكرر (على الأقلّ مرّة في الأسبوع) أقارن وزني أو شكلي أو حجمي أو عاداتي بالأكل بنساء أخريات |

1. **PHQ-4**

| خلال الأسبوعين الماضيين، كم مرة أقلقتك المشاكل التالية ؟ | أبدا | بعض الأيام | أكثر من نصف الأيام | كل يوم تقريبا |
| --- | --- | --- | --- | --- |
| 1. الشعور بالغضب أو القلق أو الانفعال الشديد | 0 | 1 | 2 | 3 |
| 2. عدم القدرة على إنهاء القلق أو التحكم فيه | 0 | 1 | 2 | 3 |
| 3. قلة الاهتمام أو قلة الاستمتاع بالقيام بأي عمل | 0 | 1 | 2 | 3 |
| 4. الشعور بالحزن أو الاكتئاب أو اليأس | 0 | 1 | 2 | 3 |

1. **مؤشّر بيتسبرغ لجودة النوم**

| **التعليمات: تتعلّق الأسئلة التالية بعادات النوم لديك على مرّ الشهر الماضي فقط. ينبغي لإجاباتك أن توفّر المعلومات الأكثر دقة لمعظم الأيام والليالي خلال الشهر الماضي. يرجى منك الإجابة عن جميع الأسئلة. خلال الشهر الماضي،** |
| --- |
| 1. في أيّ ساعة كنت تخلد للفراش ليلاً؟ وقت النوم المعتاد: _____________________ |
| 1. كم من الوقت (بالدقائق) يمرّ قبل أن تغفو كلّ ليلة؟ عدد الدقائق: _________________________ |
| 1. في أي ساعة تستيقظ صباحاً؟ وقت الاستيقاظ المعتاد: _________________________ |
| 1. أ. ما عدد الساعات التي تقضيها فعلياً في النوم ليلاً ؟ __________________ 2. ب. ما عدد الساعات التي تقضيها في السرير؟ ____________________ |

التعليمات: في كلٍ من الأسئلة المتبقية، ضع علامةً بجانب الإجابة الأفضل. يرجى الإجابة عن الأسئلة كافة.

1. خلال الشهر الماضي، كم مرةً وجدت صعوبةً في النوم لأنّك...

|  | | | ليس خلال الشهر الماضي (0) | أقلّ من مرة في الأسبوع (1) | | مرة أو مرتين في الأسبوع (2) | | ثلاث مرات أو أكثر في الأسبوع (3) |
| --- | --- | --- | --- | --- | --- | --- | --- | --- |
| أـ لا تتمكن من النوم في غضون ثلاثين دقيقة | | |  |  | |  | |  |
| ب. تستيقظ في منتصف الليل أو في الصباح الباكر | | |  |  | |  | |  |
| ج. تستيقظ لتدخل الحمّام | | |  |  | |  | |  |
| د. تعجز عن التنفّس بشكلٍ طبيعي | | |  |  | |  | |  |
| ه. تنتابك نوبات السعال أو تشخر بصوتٍ عالٍ | | |  |  | |  | |  |
| و. تشعر بالبرد الشديد | | |  |  | |  | |  |
| ز. تشعر بالحرّ الشديد | | |  |  | |  | |  |
| ح. ترى الكوابيس | | |  |  | |  | |  |
| ط. تشعر بالألم | | |  |  | |  | |  |
| ي. أسباب أخرى، يرجى التوسّع فيها، بما في ذلك ما تواتر تكرار عدم تمكنك من النوم بسبب ذلك | | |  |  | |  | |  |
|  | | | ليس خلال الشهر الماضي (0) | أقلّ من مرة في الأسبوع (1) | | مرة أو مرتين في الأسبوع (2) | | ثلاث مرات أو أكثر في الأسبوع (3) |
| 6. خلال الشهر الماضي، كم مرة تناولت الأدوية (بناءً على وصفة طبيب أو تلك المتوافرة من دون وصفة) لمساعدتك على النوم؟ | | |  |  | |  | |  |
| 7. خلال الشهر الماضي، كم مرة وجدت صعوبةً في البقاء مستيقظاً أثناء القيادة، أو تناول الطعام، أو المشاركة في نشاط اجتماعي؟ | | |  |  | |  | |  |
| 8. خلال الشهر الماضي، لأيّ مدى وجدت مشكلةً في أن تحافظ على حماستك حيال إنجاز أمور معينة؟ | | |  |  | |  | |  |
|  | جيّدة جداً (0) | جيّدة إلى حدّ ما (1) | | | سيّئة إلى حدّ ما (2) | | سيّئة جداً (3) | |
| 9. خلال الشهر الماضي، كيف تقيّم جودة النوم لديك إجمالاً؟ |  |  | | |  | |  | |

1. **BDS-10**

| كثيرا(3) | باعتدال (2) | قليلا (1) | أبدا (0) | **خلال الشهر الماضي** |
| --- | --- | --- | --- | --- |
|  |  |  |  | 1- ينقلب مزاجي بسرعة |
|  |  |  |  | 2- أغضب لأتفه الأسباب |
|  |  |  |  | 3- أفكاري مشوّشة |
|  |  |  |  | 4- أنعزل عن الاخرين |
|  |  |  |  | 5- لديّ صعوبة في التركيز |
|  |  |  |  | 6- لدي صعوبة في الاسترخاء |
|  |  |  |  | 7- أعاني مشاكل في الذاكرة |
|  |  |  |  | 8- أعاني كتمان المعدة أو الإسهال |
|  |  |  |  | 9- أشعر بشيء يمسك معدتي |
|  |  |  |  | 10- أشعر بحرقة في معدتي |

**خصائص الحمل**

1. **ما هي مدة حملك من حيث الأسابيع أو الأشهر؟** ______________ أسابيع أو __________ شهرًا
2. **هل هذا حملك الأول؟** ☐ لا ☐ نعم
3. **إذا لم يكن هذا حملك الأول ، فيرجى يرجى ذكر عدد حالات الحمل السابقة** ______________
4. **كم عدد الأجنة التي تحملينها؟** ☐1 ☐2 ☐3 أو أكثر
5. **ما هو جنس الجنين؟** ☐ذكر☐ أنثى ☐لا أعرف
6. **هل تعانين من أي مضاعفات في الحمل؟ [ضعي علامة على كل ما ينطبق]**

☐ لا شيء ☐ الإمساك ☐فقر الدم ☐ارتفاع ضغط الدم ☐ غير ذلك ، حدد ________

1. **هل تعانين من سكري الحمل؟** ☐ خضعت لفحص السكري ولا أعاني منه ☐ نعم ☐ لم أخضع لفحص السكري بعد
2. **ما هو طولك؟** ___________
3. **ما كان وزنك قبل الحمل؟** _______________
4. **ما هو وزنك الحالي؟** _______________
5. **هل تتناولين أي مكملات غذائية؟ [ضعي علامة على كل ما ينطبق]**

لا ☐ نعم، الحديد ☐ الكالسيوم فيتامين ب 9 اليود ☐ فيتامينات متعددة ☐ مكملات أخرى ، حددي _______________

1. **الرعاية الصحيّة الخاصّة بكِ ؟** ☐ طبيب خاص ☐ طبيب ، رعاية صحية أولية ☐قابلة ☐ مقدم رعاية آخر ، حددي: ___________
2. **هل تنوين الإرضاع؟** ☐ نعم ، أنوي الرضاعة حصرا ☐ نعم ، أنوي الرضاعة بالإضافة لحليب الأطفال ☐ لا ☐ لم أقرر بعد

**الخصائص الاجتماعية الديموغرافية**

1. **كم عمرك؟** ___________
2. **أين تعيشين؟** بيروت جبل لبنان الشمال البقاع الجنوب
3. **هل تعيشين في مدينة أم قرية؟**  مدينة قرية
4. **المستوى التعليمي؟** أمية ابتدائي متوسط ثانوي/ ما يعادله جامعي/ ما يعادله
5. **الوضع الاجتماعيّ ؟** عزباء متزوجة في علاقة متينة، غير متزوجة أرملة مطلقة

**المهنة؟** غير موظفة  موظفة، طوال الوقت، حددي: ___________ موظفة جزئيا، حددي: ___________

1. **حدّدي نوع الضمان أو التّأمين الّذي يشملك؟ [ضعي علامة على كل ما ينطبق]**

لا أحد  التأمين الخاص  صندوق الضمان الإجتماعي  تعاونية موظفي الدولة  الجيش  طرف اخر، حددي ___________

1. **هل تدخنين السجائر حاليا؟** لا نعم، حددي: ___________سيجارة /في اليوم
2. **هل تدخنين النارجيلة حاليا؟** لا نعم
3. **إذا كنت تدخنين النارجيلة حاليا ، فكم مرة؟**  أقل من نرجيلة واحدة أو واحدة في اليوم )بين 10 و 30 دقيقة عدد قليل = من النفخات( أكثر من واحدة في اليوم ، حدّدي عدد الدقائق
4. **هل تتعرضين للتدخين السلبي، أي الاستنشاق اللاإرادي لدخان سج أو نارجيلة الآخرين...؟** لا نعم

**الخصائص المالية**

1. **كم عدد الأشخاص الذين يعيشون في البيت، بما فيهم أنت؟** ___________
2. **ما هو عدد الغرف في البيت؟** ___________
3. **ما هو مدخول عائلتك الشهري بالدولار؟** ___________
4. **هل أنت مستقلة ماديا؟** نعم لا  افضل أن لا أقول
